# Genomic diversity of the African malaria vector *Anopheles funestus*

**DOI:** 10.1101/2024.12.14.628470

**Published:** 2024-12-17

**Authors:** Marilou Boddé, Joachim Nwezeobi, Petra Korlević, Alex Makunin, Ousman Akone-Ella, Sonia Barasa, Mahamat Gadji, Lee Hart, Emmanuel W. Kaindoa, Katie Love, Eric R. Lucas, Ibra Lujumba, Mara Máquina, Sanjay Nagi, Joel O. Odero, Brian Polo, Claire Sangbakembi, Samuel Dadzie, Lizette L. Koekemoer, Dominic Kwiatkowski, Erica McAlister, Eric Ochomo, Fredros Okumu, Krijn Paaijmans, David P. Tchouassi, Charles S. Wondji, Diego Ayala, Richard Durbin, Alistair Miles, Mara K. N. Lawniczak

**Affiliations:** 1 Wellcome Sanger Institute, Hinxton, United Kingdom; 2 CIRMF, Franceville, Gabon.; 3 Kenya Medical Research Institute, Kenya; 4 Centre for Research In Infectious Disease, Yaounde, Cameroon; 5 Ifakara Health Institute, Tanzania; 6 Liverpool School of Tropical Medicine, United Kingdom; 7 Centro de Investigação em Saúde de Manhiça (CISM), Maputo, Mozambique; 8 Institut Pasteur de Bangui, Gabon; 9 Noguchi Memorial Institute for Medical Research, Legon, Ghana; 10 University of the Witwatersrand, Johannesburg, South Africa; 11 Natural History Museum, London, United Kingdom; 12 Arizona State University, Arizona, United States; 13 International Centre of Insect Physiology and Ecology, Nairobi, Kenya; 14 MIVEGEC, Univ. Montpellier, CNRS, IRD, Montpellier, France; 15 Institut Pasteur, Antananarivo, Madagascar; 16 University of Cambridge, Cambridge, United Kingdom

## Abstract

*Anopheles funestus* s.s. is a formidable human malaria vector across sub-Saharan Africa. To understand how the species is evolving, especially in response to malaria vector control, we sequenced 656 modern specimens (collected 2014–2018) and 45 historic specimens (collected 1927–1967) from 16 African countries. We find high levels of genetic variation with clear and stable continental patterns. Six segregating inversions might be involved in adaptation of local ecotypes. Strong recent signals of selection centred on canonical insecticide resistance genes are shared by multiple populations. A promising gene drive target in *An. gambiae* is highly conserved in *An. funestus*. This work represents a significant advance in our understanding of the genetic diversity and population structure of *An. funestus* and will enable smarter targeted malaria control.

## Main text:

**Fig. 1.**
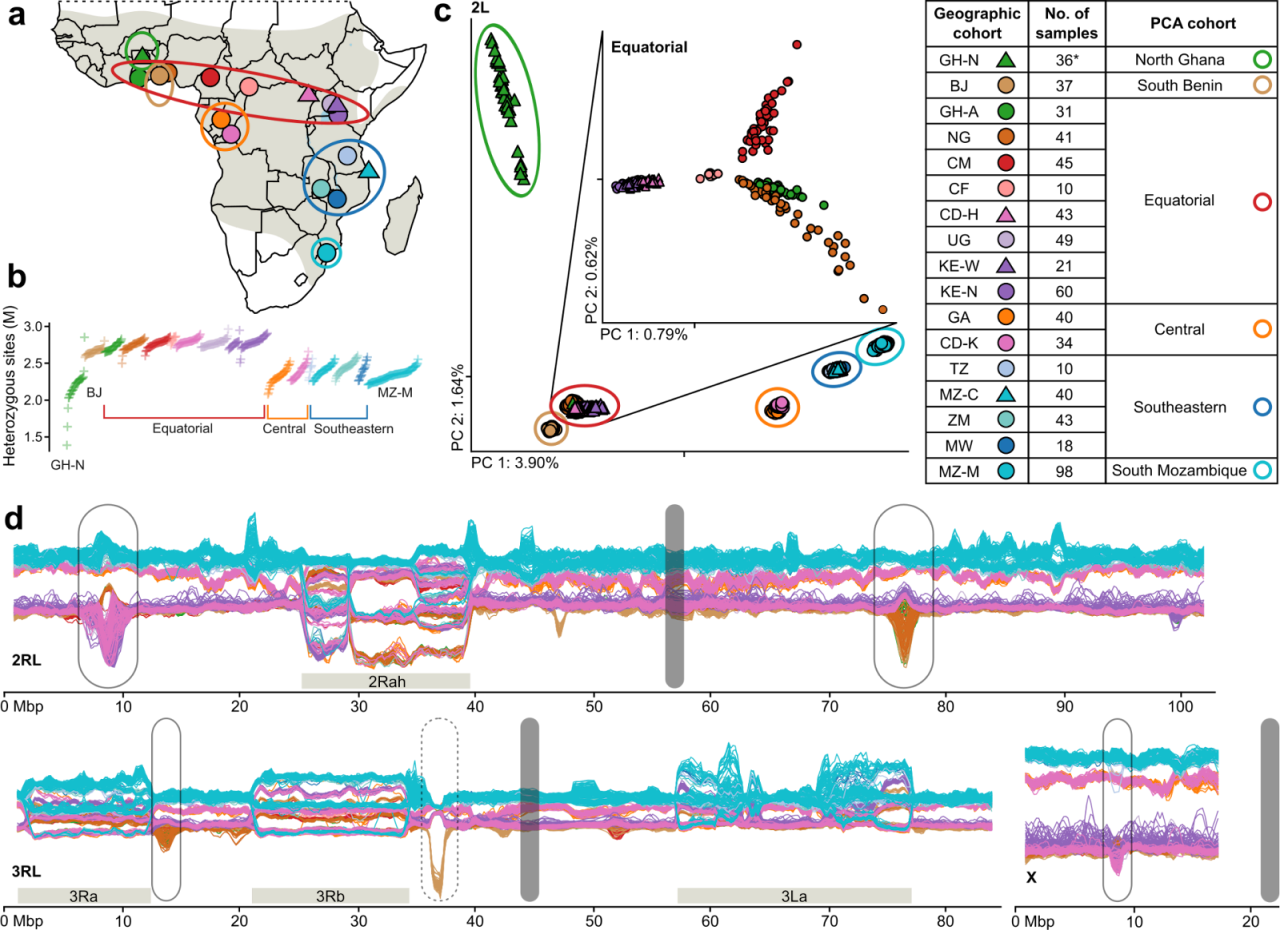
Population structure of 656 *Anopheles funestus* specimens collected across Africa. (**a**) Map showing sequenced individuals from sub-Saharan Africa grouped into 17 geographic cohorts based on their collection location (filled circular and triangular markers), and six PCA cohorts consisting of one or more geographic cohorts (open elliptical markers), with the grey shaded area showing the *An. funestus* range across the continent (adapted from Sinka et al. (1)). (*) One sample collected in GH-N clusters with Equatorial on the PCA and has therefore been removed in analyses using PCA-cohorts. (**b**) Number of heterozygous sites (in millions) per individual, with each cross representing one individual in subset_2 (females only, for subset_1 see fig. S2a) and ordered along the horizontal axis by geographic cohort (colour, same order as the legend) and number of heterozygous sites. (**c**) Projection along the first two principal components (PCs) computed on chromosome arm 2L, with the percentage of explained variance in the axis labels, and ticks denoting the 0 position along each axis. The PCA cohorts are indicated with open elliptical markers. The inset PC plot was computed only on samples from the Equatorial PCA cohort. (**d**) Sliding window PCA along the genome, each line corresponds to one individual, individuals from GH-N are excluded. The genome is split in overlapping windows of approximately 1 Mbp and 200 kbp steps, a PCA is performed for each window, PC1 values are plotted along the y-axis, and the window centres in genomic coordinates are plotted along the x-axis. (The corresponding plot displaying the PC2 values for the same windows is shown in fig. S5a). Inversion regions are indicated by horizontal grey bars, centromeres by vertical grey bars, lined ellipses indicate regions of divergence across several cohorts (Fig. 3, figs. S6–8), while the dotted ellipse showcases divergence specific to the South Benin PCA cohort (fig. S3c,d).

The mosquito species *Anopheles funestus* (Giles, 1900) has an extraordinary adaptive potential demonstrated by its vast geographic range that spans sub-Saharan Africa (1) (shaded region in Fig. 1a). Importantly, *An. funestus* is highly anthropophilic (2), has a significantly longer lifespan than other African malaria vectors (3), and in some areas is also reported to have extremely high *Plasmodium* infection rates (4–6). In much of eastern and southern Africa, the species is the major malaria vector (7). Like the vectors in the Gambiae Complex, the species is contributing to outdoor, early evening and late morning biting in response to the use of indoor-based interventions, including bed nets (8–10). Although it can be difficult to find as larvae, it may have an extended transmission season in some locations, probably due to a preference for larval habitats that persist in the dry months (11). *An. funestus* harbours several polymorphic chromosomal inversions that play an important role in adaptation (12). Similar to the three other major African human malaria vectors, which are all members of the Gambiae Complex, *An. funestus* exhibits insecticide resistance across its range (13) and in some locations, even higher tolerance to insecticides than *An. Gambiae* (14). Alleles that confer resistance can be shared across great distances or can differ locally, indicating a complicated mix of population connectivity and selective pressures (15). These characteristics, combined with its widespread distribution, make *An. funestus* a critical target for malaria control efforts. Substantial genomic data resources exist for the three major malaria vector species of the Gambiae Complex (16) and these have become foundational for the study and implementation of control efforts (17). Here, we establish a baseline understanding of genetic diversity, population structure, inversion frequencies, and historic and current selection in *An. funestus* across the African continent at a whole genome level.

### Sample acquisition, sequencing, and genetic diversity

In 2017, we put out an open call to join the project through contributing wild *An. funestus* samples collected from 2014 onwards. We carried out short read sequencing at ~35x coverage depth for over 800 wild caught specimens, of which 656 individuals from 13 African countries passed all quality controls (Fig. 1a, fig. S1a, and Methods). Sequencing reads were aligned to a 251 Mbp high quality chromosomal reference genome created from a wild caught individual from Gabon (18), and each successfully sequenced sample was assigned to a geographic cohort based on its original collection location (Fig. 1a, table S1). Using a static-cutoff (sc) site filter, 73 million (M) out of 162 M (45%) accessible sites are segregating among the sequenced samples (fig. S1d, Methods). Disregarding singletons, 49 M single nucleotide polymorphisms (SNPs) are present on two or more chromosomes, 17.3% of which have more than two alleles. For some analyses, we use a more stringent decision-tree (dt) site filter that results in 48 M out of 114 M (42%) segregating accessible sites, with 31 M present on two or more chromosomes, and 15.2% having more than two alleles. Genome-wide nucleotide diversity is between 1.4% and 1.7% in each geographic cohort (fig. S2c), with most individuals having between 2 and 3 million heterozygous sites, and higher diversity among equatorial individuals and cohorts (Fig. 1b and fig. S2a,c). Three mosquitoes from North Ghana have exceptionally low average heterozygosity (under 2 M variants) due to long runs of homozygosity (ROH, fig. S2b).

### Population structure

Principal Components Analysis (PCA) on variants from chromosome arm 2L (thought to have no common chromosomal inversions (19)), showed PC1 is correlated with latitude (Fig. 1c). Population structure is similar across other autosomal arms, but complicated by signals relating to segregating inversions (fig. S1e). We used the observed clusters in the PCA plot to define six PCA cohorts, five of which (North Ghana, South Benin, Central, Southeastern, and South Mozambique) contain relatively proximal geographic cohorts, whereas the sixth Equatorial cohort includes samples from seven countries spanning a 4,000 km range from Ghana to Kenya (Fig. 1a,c), suggesting a high degree of genetic connectivity across the equatorial region of Africa. Further PCA on the Equatorial cohort alone shows that the samples are clustered by geographic location (Fig. 1c, inset). Although the North Ghana and South Benin cohorts belong to *An. funestus* (fig. S3a) and are geographically close to the Equatorial cohort, they are genetically distinct (explored in more detail below). We also explored the mitochondrial genome for every individual, finding that the mitochondrial tree is largely consistent with the PCA cohorts (fig. S9e), although ten geographic cohorts have a small number of individuals falling in a separate mitochondrial lineage than the majority, and more than half of the South Mozambique samples are placed in ‘Lineage II’, which also contains other species from the Funestus Subgroup (20, 21).

Samples from the Equatorial cohort display more negative Tajima’s D values than those from other PCA cohorts supporting an expanding population (fig. S2d). Pairwise fixation indices (F_ST_) and patterns of doubleton sharing support the PCA cohort groupings (fig. S2e,f). PCA was computed on common variants (minor allele frequency >0.01) that tend to be older, whilst doubleton sharing focuses on rare variants that tend to be more recent (22), suggesting that the observed structure does not differ considerably between historic and recent events. While further sampling across the continent is needed, these results indicate that there is one genetically connected population of *An. funestus* across the equator, while other populations both geographically proximal and distant to the Equatorial cohort are much more genetically isolated.

### Ecotypes and differentiated populations

Our understanding of *An. funestus* ecotypes has been enhanced through population genomic sequencing of Kiribina and Folonzo, which are considered chromosomal forms and were originally characterised by non-random association of inversion karyotypes (23, 24). This recent work showed genome wide differentiation between these sympatric ecotypes within Burkina Faso. The continentally distributed Folonzo ecotype has varying inversion frequencies and a preference to breed in vegetative ditches and swamps, while the geographically restricted Kiribina ecotype has a nearly fixed standard karyotype and has adapted to breed in rice fields (23, 24). Integrating these previously sequenced specimens into our dataset, we find that outside the inversion regions, Folonzo clusters with the Equatorial cohort and Kiribina is genetically distinct from Folonzo (fig. S3b), consistent with previous findings (23). However, two cohorts we investigate here that are geographically proximal to the equator – North Ghana and South Benin – are even more genetically distinct from Folonzo than Kiribina is.

The North Ghana cohort has reduced levels of diversity, an excess of ROHs, and high divergence from all other cohorts, including from GH-A, less than 400 km away, consistent with a recent population bottleneck (Fig. 1b, figs. S2, S3d). Comparing the two populations from Ghana reveals elevated F_ST_ in the genomic regions associated with the 2R and 3L inversions (fig. S3e) and indeed, North Ghana appears to be an outlier for its 2R karyotype in comparison to its nearest neighbours (fig. S4e). We were also unable to karyotype North Ghana for the 3La inversion, due to high divergence on the 3L arm particularly in the inversion region (fig. S3e,f). One individual from North Ghana actually clustered with the GH-A population. Together, these karyotypic anomalies alongside a potentially sympatric population that looks to be Folonzo hint that the North Ghana cohort may be another ecotype, but further sampling will be needed to confirm this hypothesis.

The South Benin (BJ) cohort has similar levels of variation as the Equatorial cohort but is clearly differentiated in the PCA (Fig. 1c). To further explore this differentiation, we ran sliding window PCAs that result in a view of population structure along the genome (Fig. 1d, doi.org/10.5281/zenodo.13993020 for interactive plots; North Ghana was excluded from these analyses due to excessive ROH). While BJ follows the Equatorial cohort for most of the genome, it is highly differentiated from all other geographic cohorts in one genomic region (3R: 37Mb). F_ST_ between BJ and the neighbouring GH-A shows the strongest differentiation between the two populations falls between two genes characterised as ‘semaphorin-2A-like’ (AFUN2_001563, AFUN2_006509) (fig. S3c). We do not find any clear nonsynonymous differentiation between these populations and it may be that the divergence is regulatory in nature or that the reference genome is inadequately representing the sequences of these individuals in this genomic region (table S3). Populations from Benin are exceptionally resistant to DDT (0% mortality after one hour of exposure) (25), but there is currently no evidence in the literature to support a role in DDT resistance for semaphorin-2As, which are secreted or transmembrane signalling proteins; instead they have been implicated in nerve development, limb development, and olfaction (26, 27). Strong divergence could be caused by differential regulation of genes involved in DDT resistance, assortative mating, or habitat adaptation, but genome accessibility drops off as F_ST_ increases and the cohort is too diverged from the reference genome in this genomic region to speculate further.

**Fig. 2.**
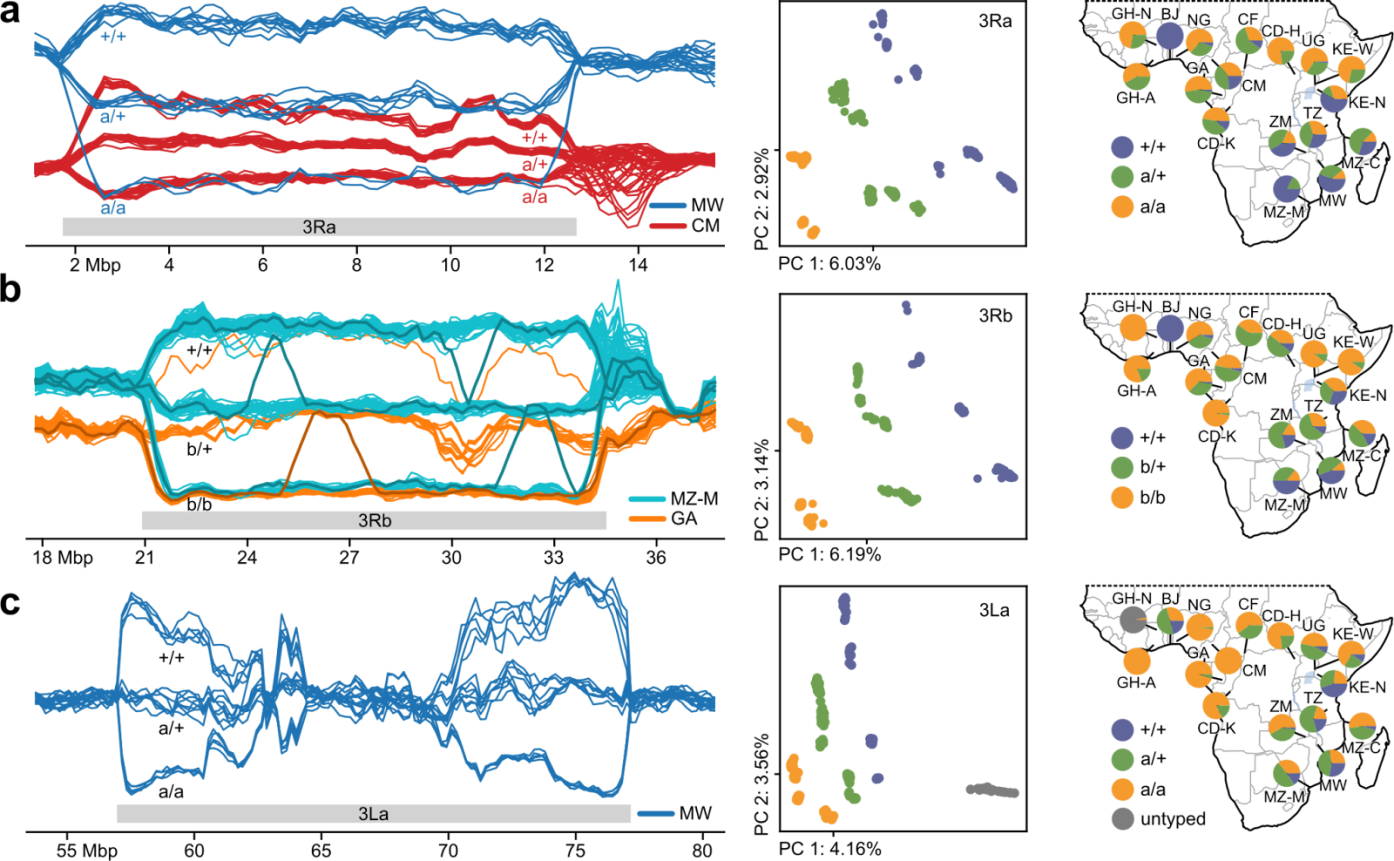
Segregating inversions on chromosome 3. Sliding window PCAs (left panels) were computed on all samples except North Ghana. For visualisation purposes, only a subset of samples is shown in the sliding window PCAs. (**a**) (left) Sliding window PCA showing CM (red) and MW (blue) samples on the 3Ra inversion region; window centres in Mbp along the x-axis, PC1 values for each individual along the y-axis. The different karyotypes are visible as three different horizontal trajectories, from top to bottom 3R+/+, 3Ra/+, and 3Ra/a. All three karyotypes are present in both cohorts, and because PC1 captures a signal that is a combination of the inversion and geographic population structure, the upper and middle trajectories (3R+/+ and 3Ra/+, respectively) do not overlap for the different cohorts, see fig. S5d. (middle) Projection along the first two principal components computed on the entire inversion region using all samples; samples are coloured by inversion karyotype. (right) Map of karyotype frequencies per geographic cohort. (**b**) (left) Sliding window PCA on the 3Rb inversion region displaying GA (orange) and MZ-M (cyan). Several samples change trajectory for part of the inversion region (indicated by thicker, darker lines); these samples appear to be double recombinants that locally exhibit a different karyotype than they have for the rest of the inversion (see fig. S5e, Supplementary Information). (middle and right) as in (a). (**c**) Sliding window PCA displaying MW on the 3La inversion region shows a strong separation of karyotypes near the breakpoints, but the signal decays towards the inversion centre (see fig. S5b). (middle and right) as in (a).

BJ has inversion frequencies that are also strongly contrasting with its close geographic neighbours: it is fixed standard for all inversions except 3La (Fig. 2, fig. S4e). This is reminiscent of the Kiribina ecotype, which is (nearly) fixed standard for 2Ra, 3Ra and 3Rb (23, 24), however, BJ is clearly differentiated from Kiribina (fig. S3b). Furthermore, in this sample set, we find no evidence of a Folonzo-like population in South Benin, so it is unclear whether BJ is sympatric with Folonzo or is the only form present in the region.

### Inversions

Chromosomal inversions can capture coadapted alleles and protect them from recombination (28). In the sliding window PCA, we find five large segregating inversions that drive population structure in their respective genomic regions, and after determining inversion breakpoints we identified these inversions as 2Ra, 2Rh, 3Ra, 3Rb, and 3La (Fig. 1d, fig. S1b, Supplementary text, table S2). We performed *in silico* karyotyping for all samples and all inversions using sliding window and aggregated PCA on the inversion region (Fig. 2, fig. S4, table S1). Inversion frequencies vary greatly across the continent and previous studies have linked inversions to behaviour and adaptation (12, 29, 30). Although Hardy-Weinberg equilibrium (HWE) of inversion karyotypes is typically satisfied within PCA cohorts, there are a few exceptions where samples collected in different seasons do not satisfy HWE when grouped together (Supplementary text). Changes in inversion frequencies during the wet and dry seasons have previously been reported for *An. funestus* (31) as well as the Gambiae Complex (32). The inversions also differ in heterozygosity of each homokaryotype: 2Rh/h has lower heterozygosity than 2R+^h^/+^h^, hinting that the standard orientation is likely ancestral (fig. S5d).

The sliding window PCA enables the investigation of local structure within the inversions. In the genomic regions where large inversions are segregating, PC1 captures a compound signal of inversion karyotype and population structure (Figs. 1d, 2, fig. S4–5). Size, age, recombination rates, and demographic history of the inversion can all affect the relative strength of the inversion and population structure signals, and the observed amount of genetic variation in the different inversion orientations (33). While for most inversions the relative strength of these two signals is constant for the entire inversion (e.g. Fig. 2a), for 3La the inversion signal decays in the middle (Fig. 2c and fig. S5b). This distinct pattern of 3La in comparison to the other inversions may have been driven by more frequent double recombination due to its large size or due to its age. Sliding window PCA reveals individuals with inversion karyotypes that are the product of double recombination in 3Rb and 3La (Fig. 2b, fig. S5e,f). Remarkably, these double recombinants can be seen in the sliding window plots where individuals change karyotype trajectories for a part of the inversion region (Fig. 2b). Events like these move alleles from one karyotype into the other, and are important for the spread of variants (34).

Consistent with Sharakhov *et al*. (19), we observe several overlapping inversions on chromosome arm 2R: 2Ra and 2Rh, which occur in many cohorts, and 2Rt, which occurs only in the CM and CD-H cohorts (Fig. 1d, fig. S4). There are six possible combined karyotypes for 2Rah, all resulting in unique trajectories in the sliding window PCA (fig. S4b,d). The relatively rare 2Rt inversion results in two additional states in the region where it overlaps 2Ra (fig. S4c,d). We believe that the 2Rt inversion only occurs in the heterozygous state in this dataset, firstly because that results in a consistent interpretation of the sliding window PCA trajectories of the combined 2Raht karyotypes, and secondly because the samples carrying 2Rt never interpolate between two groups of homozygotes, but drive a PC by themselves. Assuming that this dataset does not contain individuals homozygous for the 2Rt inversion, both CM and CD-H are in HWE.

### Signals of selection

**Fig. 3.**
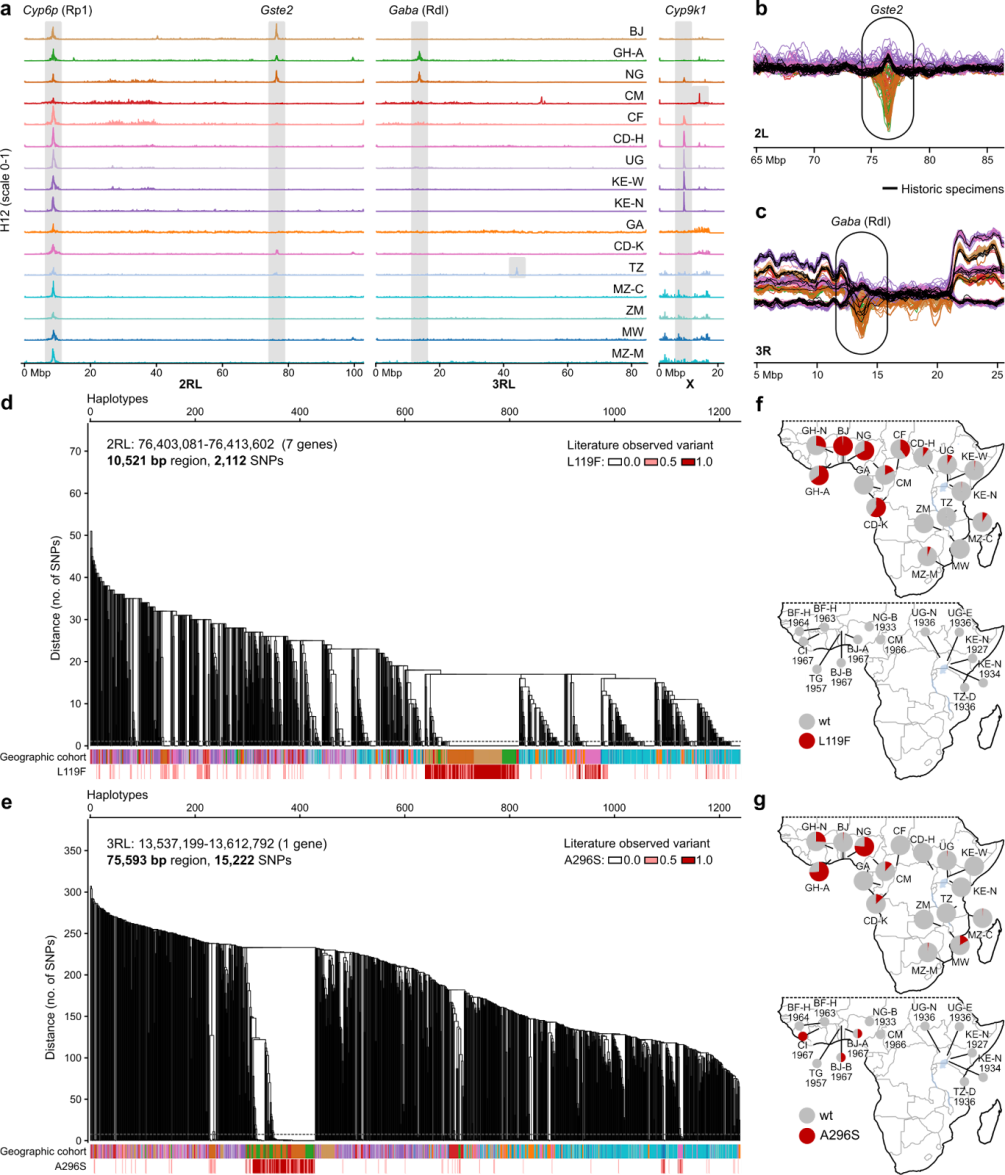
Selection sweeps and known insecticide resistance variants in *Gste2* and *Gaba*. (**a**) Genome-wide H12 scans for signals of recent selection. H12 values range from 0 to 1, with higher values indicating excessive haplotype sharing, which is a signature of recent selection. North Ghana was excluded due to elevated runs of homozygosity, which adds noise and makes H12 values unreliable for detecting genuine selection signals. The y-axis runs from 0 to 1 for each cohort, the x-axis shows positions along the genome. Peaks of H12 values ≥0.4 are highlighted with a grey vertical bar; for peaks where only one cohort reaches this threshold, the bar is restricted to this cohort. (**b**) Sliding window PCA computed on modern samples and historic samples combined, excluding North Ghana (fig. S9d). For visualisation purposes, only present-day Equatorial and South Benin cohorts and the historic Equatorial cohort are shown. Here displaying a 20 Mbp region around the *Gste2* gene. Present-day samples show a peak in the *Gste2* region, which is likely a signal of selection, while historic samples (shown in black) do not exhibit this signal. (**c**) Sliding window PCA computed and samples subsetted as in (b), here showing a 20 Mbp region around the *Gaba* gene. Several historic samples follow the peak seen in present-day samples at the *Gaba* locus. (**d**) Haplotype clustering within a region containing seven *Gste* genes. The dendrogram is obtained by hierarchical clustering of phased haplotypes, and used to define haplotype clusters as groups of haplotypes with SNP divergence below 0.0005 (cutoff indicated as dashed horizontal line on the dendrogram). The first bar below the dendrogram shows the population of origin for each haplotype, the second bar shows the genotype for the known *Gste2* L119F mutation (note that this mutation was filtered out before haplotype phasing, so each haplotype is coloured by the genotype of the individual it belongs to; fig. S6a, table S3). (**e**) Haplotype clustering within the *Gaba* gene. Same structure as in panel (d) with the red bar showing the genotype for the rdl A296S variant (fig. S6b, table S3). (**f**) Maps showcasing the frequency of the L119F mutation in present-day (above) and historic (below) sample sets. (**g**) Maps showcasing the frequency of the rdl A296S mutation in present-day (above) and historic (below) sample sets.

To explore signals of selection, we computed H12 (35), a statistic measuring haplotype homozygosity along the genome (Fig. 3a, Methods). H12 quantifies excess homozygosity indicative of hard or soft selective sweeps. We identified four regions that are putatively under very strong selection (H12 > 0.4) in at least two geographic cohorts (Fig. 3a, grey boxes); notably, these regions are also visible as peaks in the sliding window PCA (Fig. 1d, circled regions). All four H12 peaks are centred on known insecticide resistance genes that are important in many insect species (*Gste2, Gaba, Cyp6p, Cyp9k1*) (36).

The H12 peak on chromosome arm 2L, observed in BJ, GH-A, NG, and CD-K, is centred on a cluster of seven glutathione S-transferase (*Gste*) genes. The non-synonymous L119F mutation in *Gste2* is important in both DDT and pyrethroid resistance in *An. funestus* (25). We observe this mutation across the continent, at low frequencies in the east, and at particularly high frequencies in the aforementioned cohorts, notably nearly fixed in BJ (Fig. 3a,d,f, table S3). Most individuals from BJ, GH-A, and NG with the L119F mutation share the same haplotype in the *Gste* region, while in CD-K the L119F mutation is found on a different haplotypic background (Fig. 3d, fig. S6a), suggesting two distinct selective sweeps, rather than spread of a single resistance mutation.

The H12 peak on chromosome arm 3R, in GH-A and NG, is centred on the gamma-aminobutyric acid receptor subunit beta (*Gaba*) gene (Fig. 3a, table S3). In many species, a mutation at one amino acid residue in *Gaba* confers dieldrin resistance, hence being named the resistance to dieldrin (rdl) mutation (37). The A296S rdl mutation in *An. funestus* has previously been detected at high frequency in Central and Western Africa (38). The same A296S mutation is found here in ten geographic cohorts, predominantly on two distinct swept haplotypic backgrounds that occur sympatrically in GH-A and NG (Fig. 3e,g), but also spread throughout the continent at low frequency, discussed further below.

The other strong H12 peaks occurring in multiple cohorts include two other well studied insecticide resistance loci, *rp1* and *Cyp9k1*. There are also strong H12 peaks that are only found in a single cohort (TZ, CM) including a clear sweep on the well studied knock down resistance (kdr) mutation at the voltage gated sodium channel (*vgsc*) locus only in the Tanzanian samples – these samples were collected from a region that has a DDT stockpile that was leaking into the environment for decades (39). These loci are all discussed in the Supplementary text and figs. S7–8.

It is clear that some of the strongest selective pressures observed in this species are driven by insecticides. Widespread use of synthetic insecticides began in Africa in the late 1940s with the advent of DDT (40, 41). To begin to explore both when and where the mutations we observe above arose, we sequenced 75 historic mosquitoes labelled as *An. funestus* from museum collections spanning 1927–1973 (fig. S9, table S4, Methods) (42). Based on mitochondrial and nuclear sequencing data, only 45 of these (spanning 1927–1967) were actually *An. funestus* s.s. (table S4). These 45 samples originated from nine countries and clustered together with the Equatorial and Southeastern PCA cohorts, which is expected based on their origins (fig. S9c). We integrated the historic data into the sliding window PCA, which showed that most of the genome in historic samples followed their present-day cohorts well, suggesting that population structure has been relatively stable over the past century (fig. S9d, see doi.org/10.5281/zenodo.13993020 for interactive plots). Intriguingly, nine historic samples follow the trajectory of South Benin in the divergence peak in chromosome arm 3R, and the alleles observed in the historic samples are highly similar to those found in modern day South Benin (fig. S10). In South Benin, there is exceptionally strong DDT resistance (43) but it remains to be determined what this allele, which is now found only in South Benin, was associated with in these historic populations.

Emerging DDT resistance was phenotypically detected in *An. funestus* in the late 1950s (44), however, we do not find any evidence of either L119F in *Gste* or kdr in *vgsc* that are associated with DDT resistance today (table S4). We suspect that if the kdr mutation conferred resistance to DDT 60 years ago, it would have been rapidly reselected when pyrethroid treated bed nets were distributed at scale. Historic genomes also do not have evidence of insecticide resistance mutations at *rp1* or *Cyp9k1* (table S4), suggesting that selection pressures on these genes are more recent. However, we find that six individuals from the 1960s, the time when dieldrin resistance in *An. funestus* was first reported (44, 45), follow the PC1 trajectory of present-day resistant populations that carry the rdl A296S mutation (Fig. 3b, fig. S9d) and indeed each of those individuals carry the mutation in a heterozygous or homozygous state. *Gaba* might act as a secondary target for pyrethroids (46), which could explain the persistence of rdl in modern populations. Alternatively, or additionally, dieldrin, like DDT, is a persistent organic pollutant and also has stockpiles across Africa. Perhaps leaking dieldrin stockpiles continue to exert selective pressure on modern populations or perhaps unregulated usage continues in some areas. Undoubtedly, these long lasting insecticides that are stored in poor conditions in many locations across Africa are complicating resistance management and vector control (47).

Today, while we observe that both the L119F mutation in *Gste2* and the rdl mutation in *Gaba* are on haplotypes undergoing selective sweeps in west and central Africa, they are also found scattered across the continent at lower frequencies (Fig. 3d-g). This may hint that historic selection for these mutations was relaxed during the period of the 1970s-1990s when DDT and dieldrin usage declined, but these mutations may have remained at low frequencies, preadapting the species to rapidly evolve resistance to pyrethroids when those came into common usage in the 2000s.

### Gene drive

The use of gene drive to eliminate or modify malaria vector species holds great promise as a targeted malaria vector control strategy (48). Knowledge of genetic variation and structure throughout the species range is important to both assess the likelihood of geographical spread and to identify candidate targets for CRISPR-Cas9 gene drive, given that polymorphism within target sites affects gene drive efficacy (16). Suppression based gene drive targeting the doublesex (*dsx*) gene has already been developed and successfully tested in the laboratory and large cage setting for *An. gambiae* (49, 50). Approaches developed for *An. gambiae* should be technically sound for *An. funestus*, but population structure and levels of genetic diversity will differ between these species.

**Fig. 4.**
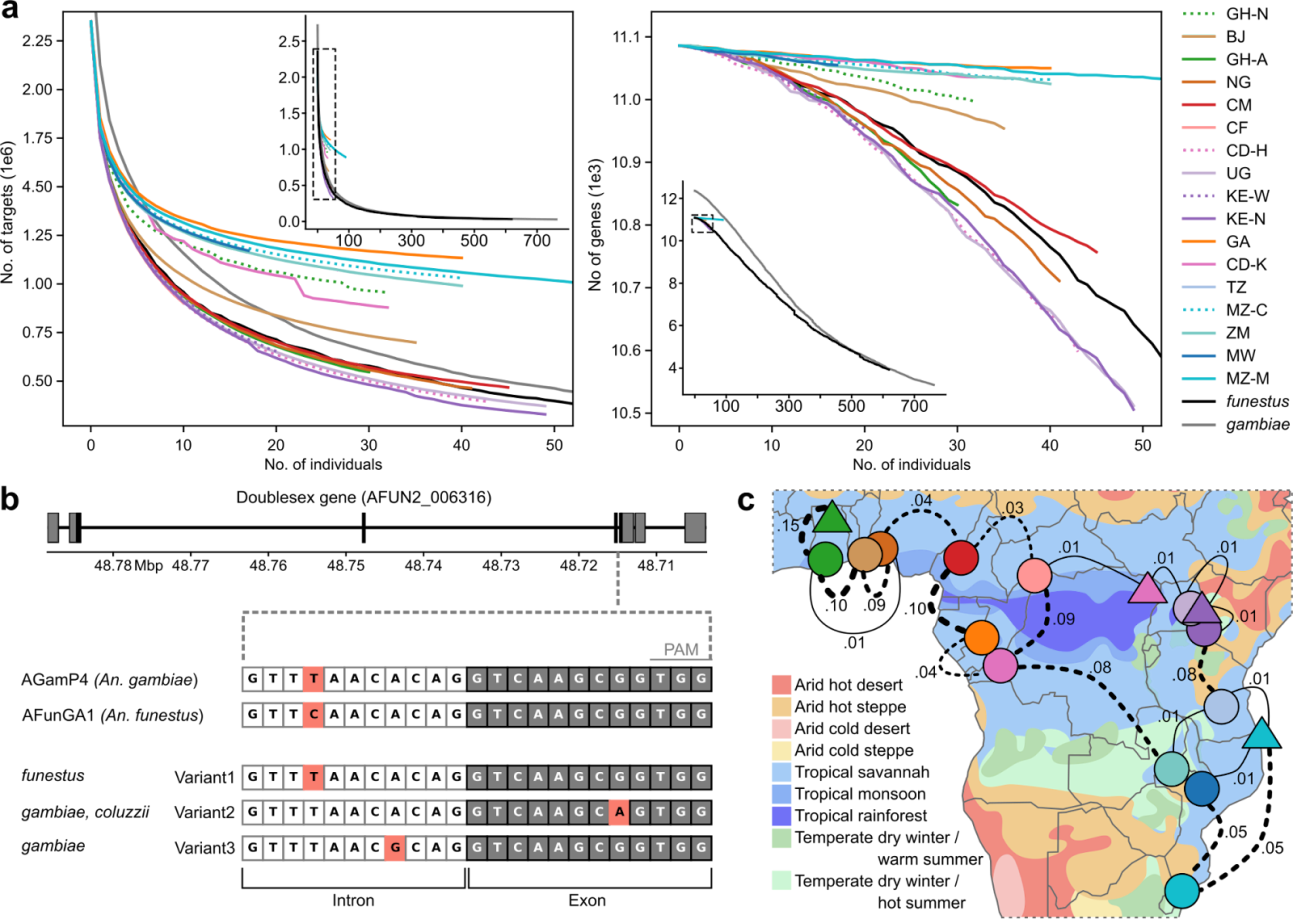
Challenges for vector control. (**a**) Cumulative number of gene drive targets (left) and number of genes containing gene drive targets (right) per geographic cohort as well as all subset_2 individuals (*funestus*) and *An. gambiae* and *An. coluzzii* individuals from Ag1000G phase 1 (16) (*gambiae*). The insets are zoomed out versions of the main plot, showing the results for the fully explored datasets; the areas of the main plots are indicated as dashed boxes in the insets. (**b**) Sequence variation in the *dsx* gene drive target on chromosome arm 2R. The *dsx* gene drive target is located at the boundary of intron 4 and exon 5 of the female-specific isoform. At the top, the seven exons of the female-specific isoform are depicted as boxes on the AGamP4 reference genome; coding sequence is shown in black, untranslated exonic regions (UTR) in grey. The target sequence is shown below, nucleotides in white boxes are located in intron 4, nucleotides in grey boxes are located in exon 5; the last three nucleotides constitute the PAM. The AGamP4 (*An. gambiae*) and AFunGA1 (*An. funestus*) reference genomes differ at the fourth base of the target sequence (highlighted in pink) in the intronic region. Variant 1 is found at very low frequencies in two cohorts from this study, Variant 2 is found at low to intermediate frequencies in four cohorts of *An. gambiae* and *An. coluzzii* and Variant 3 is found at very low frequency in one cohort of *An. gambiae* (see Supplementary Information). (**d**) Map showing fixation indices (F_ST_) for neighbouring populations. F_ST_ is calculated on all accessible sites on the 2L chromosome arm. F_ST_ > 0.01 lines are dotted and become thicker the higher the F_ST_. The background of the map is coloured by the Köppen-Geiger climate classification, adapted from Beck *et al*. (51).

Here we evaluated potential target sites within coding sequence for *An. funestus*. Gene drive targets are identified as 20 bp sequences located entirely within coding sequence, containing the protospacer adjacent motif (PAM). In total, we identified 2,349,313 target sites in 11,086 genes based on the reference genome. However, only 30,459 sites in 3,927 genes remained after excluding targets with variation in any subset_2 individual. In comparing target site availability in *An. funestus* to *An. gambiae,* we find that *An. gambiae* has more available targets based on the reference genome alone (2,718,188), but the decay in number of targets as we consider variation in cumulatively more individuals is similar to *An. funestus* (Fig. 4a, Supplementary text). We also specifically explored the *dsx* gene drive target region in *An. funestus*, finding that the reference genomes (AgamP4 and AfunGA1) only differ by one base pair (Fig. 4b), and only a single SNP is found segregating in two individuals among all the *An. funestus* we sequence here and this SNP is the *An. gambiae* reference allele. Altogether, this bodes well for using the same population suppression gene drive approach in *An. funestus* as is underway for *An. gambiae.*

## Discussion

Even if the Gambiae Complex disappeared today, malaria will still rage through Africa until *An. funestus* is also effectively targeted. The greater understanding of the high levels of genetic diversity and the complex population structure of *An. funestus* presented here will underpin smarter surveillance and targeted vector control (3, 52, 53). More than 4,000 kilometres separates the sampling sites of the Equatorial cohort (from Ghana Ashanti to Western Kenya), yet populations from across that range are genetically connected, while much geographically closer populations like South Benin and North Ghana are genetically distinct (Fig. 4c). Some of this structure may originate from geographic discontinuities like the Congo basin rainforest and the Rift valley, and some may originate from differences in climate and rainfall (51) (Fig. 4c). Small *et al.* (23) predicted that other ecotypes are likely to be found as genomic investigations into the species proceed (54, 55), and we argue that both South Benin and North Ghana may provide two more examples. The identification of these ecotypes highlights the complex population structure of *An. funestus* and the need for locally tailored control strategies.

A comprehensive understanding of gene flow within and between each of the major malaria vector species is critical for implementing effective malaria control, whether that be through gene drive release or successful insecticide use and resistance management. The high genetic diversity and complex population structure of *An. funestus* suggests that a one-size-fits-all approach to vector control may be ineffective. The identification of strong selective sweeps centred on known insecticide resistance genes across multiple populations highlights the ongoing challenge of insecticide resistance. Notably, the same mutations tend to confer resistance and be under selection in a wide range of insects (36), and in both *An. funestus* and *An. gambiae* (56) these mutations sometimes occur on multiple distinct haplotypic backgrounds suggesting populations may not be mutation limited. This convergent evolution of resistance mechanisms highlights the adaptability of these vector populations and underscores the necessity of tailored interventions that consider local genetic backgrounds and resistance profiles (57). Insecticide resistance in *An. funestus* results from a complex interplay between convergent evolution, sharing of resistance alleles between populations and changing selective pressures in space and time, and calls for the continued monitoring of resistance alleles alongside the development and deployment of novel insecticides or alternative control strategies (58). Population suppression or modification gene drives are a promising alternative to insecticide-based control and we identified a comparable number of candidate gene drive targets in *An. funestus* as in *An. gambiae* and *An. coluzzii*.

This work represents the first step in generating a foundational genomic understanding of *An. funestus* akin to what we have for the other major malaria vector species in Africa (16, 59). Population genomic data resources are increasingly critical for informing vector control and are needed to underpin both technical strategies and global health policies. Further studies will need to focus on exploring the full range of the species given the complex pattern of population connectivity, and should integrate phenotypic data to understand the functional significance of observed genotypes. Temporal sampling is a key component of vector surveillance in order to comprehend the dynamics of genetic changes, e.g. frequency changes of resistance alleles, seasonal changes in inversion frequencies, and the range and persistence of ecotypes. Beyond increased spatiotemporal sampling, long read sequencing of ecotypes and resistance alleles will enhance our understanding of gene flow in this important malaria vector.

## Supplementary Material

Supplement 1

Supplement 2

Supplement 3

Supplement 4

Supplement 5

## Data Availability

ENA accession IDs for each sample can be found in table S1 (modern) and table S4 (historic). All metadata associated with each sample is in the same tables. These data are released as part of the ENA BioProject PRJEB2141, the Vector Observatory Project. Processed data can be accessed through the malariagen_data python package at https://malariagen.github.io/vector-data/af1/af1.0.html. Analysis code will be available at github.com/malariagen/funestus_popgen_ms prior to publication.
